# Metronomic Celecoxib Therapy in Clinically Available Dosage Ablates Hepatocellular Carcinoma via Suppressing Cell Invasion, Growth, and Stemness in Pre-Clinical Models

**DOI:** 10.3389/fonc.2020.572861

**Published:** 2020-10-21

**Authors:** Chun-Chieh Yeh, Pei-Ying Liao, Sudhir Pandey, Su-Yung Yung, Hsueh-Chou Lai, Long-Bin Jeng, Wei-Chun Chang, Wen-Lung Ma

**Affiliations:** ^1^Department of Surgery, Organ Transplantation Center, China Medical University Hospital, Taichung, Taiwan; ^2^Department of Medicine, School of Medicine, China Medical University, Taichung, Taiwan; ^3^Department of Chinese Medicine, Graduate Institute of Biomedical Sciences, School of Medicine, China Medical University, Taichung, Taiwan; ^4^Sex Hormone Research Center, Department of Gastroenterology, China Medical University Hospital, Taichung, Taiwan; ^5^Sex Hormone Research Center, Department of OBS & GYN, China Medical University Hospital, Taichung, Taiwan; ^6^Department of Nursing, Asia University, Taichung, Taiwan

**Keywords:** NSAID (nonsteroidal anti-inflammatory drug), Celecoxib, hepatocellular carcinoma (HCC), NFκB, metronomic, chemoprevention

## Abstract

**Objective:**

To investigate the anti-carcinogenic effect of metronomic Celecoxib (i.e., frequent administration in clinically available doses) against hepatocellular carcinoma (HCC) in the perspective of metastasis, spontaneous hepatocarcinogenesis, cancer invasion, proliferation, and stemness in vivo and in vitro.

**Background:**

Celecoxib, a selective cyclooxygenase-2 (COX-2) inhibitor, is known to cause anti-carcinogenic effects for HCC in suprapharmacological doses. However, the effects of metronomic Celecoxib treatment on HCC cells remain unclear.

**Methods:**

The *in vivo* chemopreventive effect of metronomic Celecoxib (10mg/kg/d) was investigated by the syngeneic HCC implantation model and spontaneous hepatocarcinogenesis in HBV-transgenic(HBVtg) mice individually. HCC cell lines were treated by either suprapharmacological (100 μM) or metronomic (4 μM) Celecoxib therapy. Anti-carcinogenic effects were evaluated using cell invasion, cancer proliferation, angiogenesis, and phenotype of cancer stem/progenitor cells (CSPC). The molecular mechanism of metronomic Celecoxib on HCC was dissected using Luciferase assay.

**Results:**

In vivo metronomic Celecoxib exerted its chemopreventive effect by significantly reducing tumor growth of implanted syngeneic HCC and spontaneous hepatocarcinogenesis in HBVtg mice. Unlike suprapharmacological dose, metronomic Celecoxib can only inhibit HCC cell invasion after a 7-day course of treatment via NF-κB/MMP9 dependent, COX2/PGE2 independent pathway. Metronomic Celecoxib also significantly suppressed HCC cell proliferation after a 7-day or 30-day culture. Besides, metronomic Celecoxib reduced CSPC phenotype by diminishing sphere formation, percentage of CD90+ population in sphere cells, and expression of CSPC markers.

**Conclusions:**

Metronomic Celecoxib should be investigated clinically as a chemopreventive agent for selected high-risk HCC patients (e.g., HCC patients after curative treatments).

## Introduction

Hepatocellular carcinoma (HCC) is the most common primary liver cancer and the 3^rd^ common cause of cancer-related mortality in the world (1). Preventive strategies for HCC are clinically relevant. They can focus on different levels, such as prevention of hepatitis B virus (HBV) related spontaneous hepatocarcinogenesis (secondary chemoprevention) and prevention of relapse or metastasis of HCC after curative treatments (tertiary chemoprevention) (2). Nearly 40% of HCC patients suffered tumor relapse within two years after curative therapies, which means a strong need for effective chemopreventive modalities (3). Prognostic factors of recurrent HCC after surgery include vascular invasion, tumor size, and expression of cancer stem/progenitor cells (CSPC) markers such as CD90 (i.e., recurrence-related CSPC marker) and CD133 (4). Thus, potential targets of chemoprevention may include cancer invasion, cell proliferation, and phenotype of cancer stem cells.

Metronomic use of chemotherapy (i.e., long-term administration at low doses without long drug-free intervals) is well known to reduce the drug-related adverse effect and the risk of developing acquired drug resistance in cancer therapy (5). Similarly, metronomic use of Aspirin and non-steroidal anti-inflammatory drugs (NSAIDs) (i.e., long-term administration at clinically available dose) are also in association with reduced risk of various cancers, including recurrent HCC after curative liver resection (6, 7). NSAIDs, particularly selective cyclooxygenase-2 (COX-2) inhibitors such as Celecoxib, could effectively inhibit cell proliferation, restore cell apoptosis, and reduce angiogenesis in various cancer cell lines (6, 8, 9). However, most of the studies were performed in the setting of using Celecoxib at suprapharmacological doses (i.e., more than 5 μmol/L) (8–11). In contrast, the anti-carcinogenic effect and relevant molecular mechanism of metronomic Celecoxib were less investigated and remained elusive.

Increased expression of COX-2 or nuclear factor-kappa B (NFκB) was in association with carcinogenesis in HCC clinically (12, 13). Celecoxib could inhibit carcinogenesis via COX-2/PGE2 dependent and independent mechanisms (6, 8). Accordingly, Celecoxib was reported to inhibit growth and induce apoptosis in HCC cells, which can be partially reversed by COX-2 and prostaglandin E2 (PGE2) treatment (14). Also, Celecoxib could reduce angiogenesis, cell division, and metastasis via nuclear factor-kappa B (NFκB)/COX-2/prostaglandins pathway or other NFκB dependent signaling pathways (e.g., NFκB/matrix metalloproteinase 9 (MMP9) or cyclin D) (8, 10). However, all these mechanisms were mainly discovered while administrating Celecoxib at supra-pharmacologic doses (8, 10, 11). By contrast, molecular mechanisms underlying metronomic Celecoxib-mediated chemoprevention against HCC recurrence remain unclear and need to be investigated. In this study, we evaluated the effects and mechanism of metronomic Celecoxib treatment in preventing recurrent HCC. We found that metronomic Celecoxib therapy suppressed tumor regrowth of implanted syngeneic HCC, spontaneous hepatocarcinogenesis in HBV transgenic (HBVtg) mice, cell invasion, proliferation, and CSPC phenotype of HCC cells in vitro. The present study filled gaps between basic and clinical studies. Moreover, metronomic Celecoxib treatment should be investigated clinically as a chemopreventive modality for selective high-risk HCC patients after curative treatments.

## Materials and Methods

### Metronomic Celecoxib Therapy on Syngeneic HCC Implantation Tumor Model and Spontaneous HBVtg-HCC Model

We followed the Guidelines for the Care and Use of Laboratory Animals (Ministry of Sciences and Technology, Taiwan) in animal experiments, which were approved by the China Medical University Committee of Laboratory Animal Welfare. We purchased Hepa1-6 cells for the syngeneic HCC model from ATCC (CRL-1830; Taipei, Taiwan), and modified the tumor development protocol from the previous report (15). We fed the mice by either placebo or metronomic Celecoxib therapy (10 mg/kg/d) 7 days earlier before Hepa1-6 (10^6^/implantation site) cells were implanted into bilateral flanks of C57BL/6 mice (n = 18 sites in metronomic Celecoxib group; n = 16 sites in placebo groups). Then, the mice received therapy consecutively for 36 days. During the treatments, we measured the body weight and subcutaneously implanted tumor size by the previous protocol (16). We sacrificed the mice on post-implant day 37 and photographed and collected the tumors.

For the spontaneous HBVtg-HCC model, we obtained the HBVtg mouse and modified HBVtg-HCC protocol from Professor James Ou at the University of Southern California (17). The HBVtg-HCC mouse model was established and characterized as described earlier. (18–20) In brief, the HBVtg mice were intra-peritoneally (i.p.) injected with a carcinogen (diethylnitroasamine; DEN; 20 mg/kg) on the 14^th^ days of pup mice. After genotyping to confirm HBVtg genotype, the mice were randomly assigned to two groups (18). For the metronomic Celecoxib group, we treated HBVtg-HCC mice with Celecoxib (10 mg/kg/daily) since the age of 20 weeks (i.e., the time of liver tumor initiation) for consecutive 16 weeks, and then sacrificed the mice at the age of 36 weeks (i.e., fast-growing phase of liver tumor) (18). During the therapy period, we recorded the bodyweight of the mice daily. The mice in the metronomic Celecoxib group (n = 6) whose body weight was comparable to those in the placebo group (n = 9) were taken to record liver weight, tumor size, and tumor number at the time of sacrifice.

### Histology Diagnosis and Immunohistochemistry

The subcutaneously implanted liver tumors from the syngeneic HCC model and whole livers from the HBVtg-HCC mice were collected and embedded in paraffine block for histology exam. The histological studies were performed with modifications as described in previous studies (16, 21). For histologic inspection, we treated tissue sections (2 μM) with hematoxylin and eosin or stained sections with antibodies specific for CD34 (abcam, ab81289) immunohistochemical (IHC) staining while using an ABC kit (Vector Laboratories) to enhance the staining signals. The slides were scanned with the Aperio ScanScope CS system (Leica Biosystems, Buffalo Grove, IL, United States) at 200× (objective lens) for further image analysis using ImageJ (NIH). The staining distributions were graded using a five-point scale according to the percentage of positive staining in whole scanned area (positive area/total area × %).

### Statistical Analysis

Statistical analyses were performed using Student’s t test. All experiments were repeated at least three times, and *P* values less than 0.05 were considered to indicate statistical significance.

The detailed materials and methods related cell culture, tube formation assay, and gene expression measurements were described in supplemental text.

## Results

### Metronomic Celecoxib Reduced *In Vivo* Tumor Regrowth of Implanted Syngeneic HCC and Spontaneous Hepatocarcinogenesis in HBVtg-HCC Models

**Figure 1:**
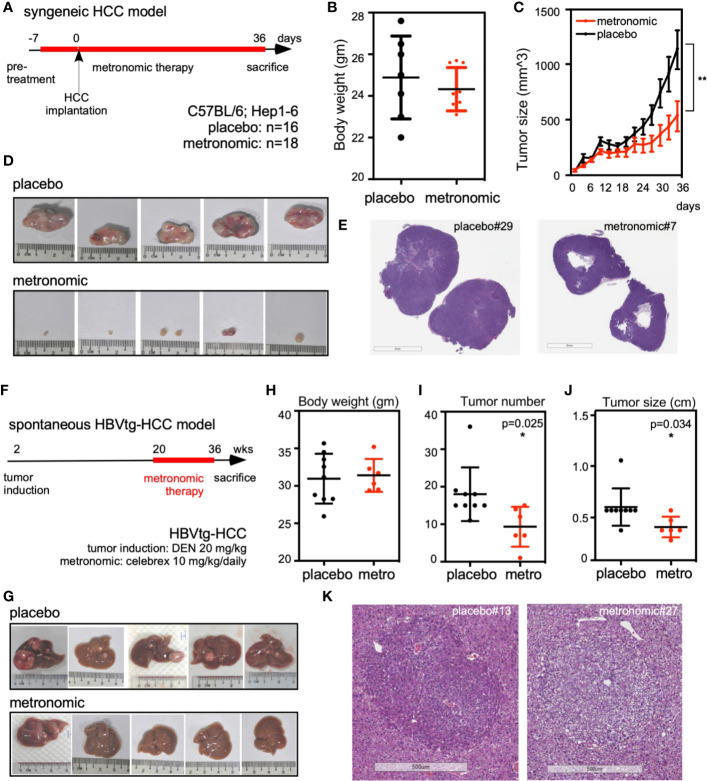
Metronomic Celecoxib significantly suppressed *in vivo* tumor regrowth of seeded syngeneic HCC and spontaneous hepatocarcinogenesis in the HBVtg-HCC model. **(A)** Protocol of metronomic Celecoxib on the syngeneic HCC implantation model. C57BL/6 mice were pretreated with metronomic Celecoxib (10 mg/kg/d) orally before implanting Hepa1-6 cells (10^6^/implantation site) into bilateral flanks. After implantation, these mice were treated with either metronomic Celecoxib or placebo for another 36 days and sacrificed on the 37^th^ day for measurement. **(B)** The bodyweight of mice was comparable between the placebo and the “metronomic Celecoxib” group. **(C, D)** The implanted Hepa1-6 HCC tumor size was significantly suppressed in the “metronomic Celecoxib” group when compared to the placebo group (day-37 tumor size [mean ± SEM] = 539.8 ± 135.8 mm^3^ vs. 1138.0 ± 175.0 mm^3^, P < 0.01). **(E)** H&E stain showed significant central necrotic portion of HCC in the “metronomic Celecoxib” group at the syngeneic HCC model. **(F)** Protocol for spontaneous hepatocarcinogenesis in the HBVtg-HCC model. HBV transgenic mice (HBVtg) mice were given Diethylnitroasamine (DEN; 20 mg/kg) intraperitoneally at the age of 14^th^ day. Metronomic Celecoxib (10 mg/kg/d) or placebo was fed from the age of 20^th^ week to 36^th^ week. Then, the mice were sacrificed for the measurement of liver tumors. **(G)** Spontaneous hepatocarcinogenesis in the harvested liver from the “metronomic Celecoxib” group was grossly less than that in the placebo group. **(H–J)** Bodyweight of mice was also comparable between the “metronomic Celecoxib” group and the placebo group. Tumor number and tumor size were significantly reduced in “metronomic Celecoxib” group compared to placebo group (tumor number [Mean ± SEM] = 9.3 ± 2.2 vs. 18.0± 2.4, P < 0.05; tumor largest diameter [Mean ± SEM] = 3.3 ± 0.4 mm vs. 5.3 ± 0.6 mm, P < 0.05). **(K)** H&E staining at comparable-sized HCCs showed less eosinophilic staining in the “metronomic Celecoxib” group compared to the placebo group in HBVtg-HCC model. * Indicates *P* < 0.05 and ** indicates *P* < 0.01.

To test the *in vivo* chemopreventive effect of metronomic Celecoxib on seeded cancer, we implanted syngeneic HCC cells into bilateral flanks of C57BL/6 mice that were fed by either metronomic Celecoxib (n = 18 sites) or placebo (n = 16 sites) as protocol (**Figure 1A**). The bodyweight of both groups was comparable that may imply metronomic Celecoxib therapy did not impair the general physiologic status of mice (e.g., growth and intake) (**Figure 1B**). However, tumor size of implanted syngeneic HCC was significantly reduced in the “metronomic Celecoxib” group compared to the placebo group (tumor volume on post-implant day 37 [mean ± SEM] = 539.8 ± 135.8 mm^3^ vs. 1138.0 ± 175.0 mm^3^, P < 0.05) (**Figures 1C, D**). H&E stating at comparable-sized HCCs showed a significant central necrosis in the “metronomic Celecoxib” group compared to the placebo group (**Figure 1E**)

To investigate the chemopreventive effect of metronomic Celecoxib on spontaneous hepatocarcinogenesis, we compared tumor number and size of HBVtg-HCC mice that were fed by either metronomic Celecoxib (n = 6) or placebo (n = 9) as protocol and harvested liver for measurement after sacrificing them (**Figures 1F, G**). The body weight of mice was comparable between both groups (**Figure 1H**). The tumor numbers were significantly reduced in the “metronomic Celecoxib” group compared to the placebo group (Mean ± SEM= 9.3 ± 2.2 vs. 18.0± 2.4, P < 0.05) (**Figure 1I**). In addition, the tumor size was also smaller in the “metronomic Celecoxib” group compared to the placebo group (tumor largest diameter [Mean ± SEM] = 3.3± 0.4 mm vs. 5.3± 0.6 mm, P < 0.05) (**Figure 1J**). H&E staining at comparable-sized HCCs showed less eosinophilic staining in the “metronomic Celecoxib” group compared to the placebo group that may imply less intracellular protein component in the metronomic group (**Figure 1K**).

### Metronomic Celecoxib Treatment During Long-Term Therapy Significantly Attenuated Cell Invasion Capability of HCC Cells

**Figure 2:**
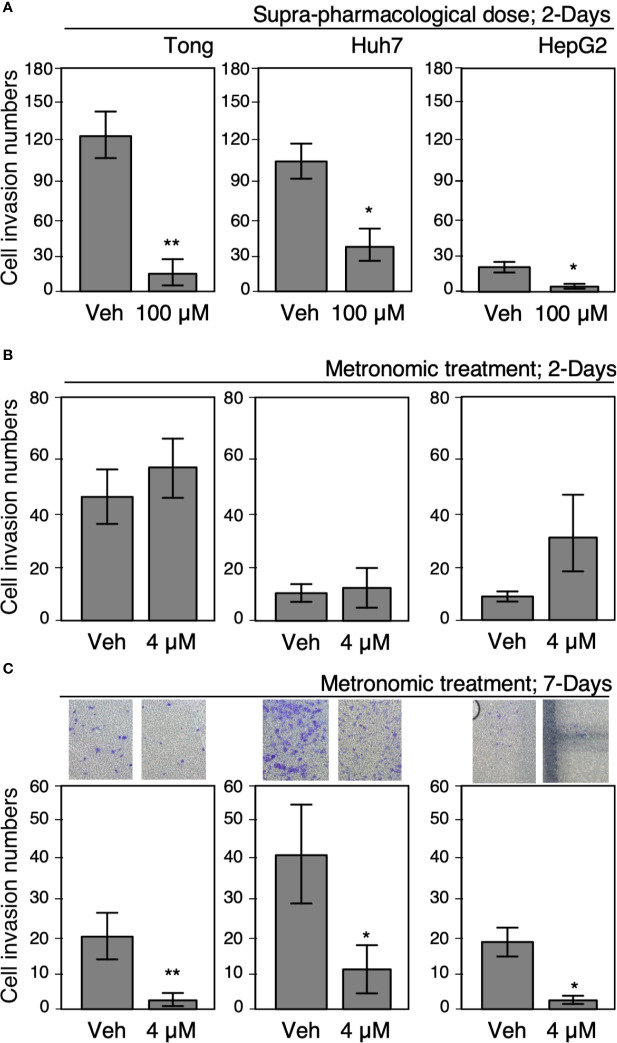
Metronomic Celecoxib treatments reduced HCC cell invasion. **(A)** Suprapharmacological Celecoxib treatments (100 μM) on human HCC cells (Tong, Huh7, and HepG2) for 2 days suppressed cell invasion. **(B)** Clinically available Celecoxib treatments (4 μM) for 2 days did not suppress cell invasion. **(C)** Metronomic Celecoxib treatment (4 μM, 7 days) could suppress cell invasion in the HCC cells. The HCC cells treated with or without Celecoxib were plated onto Matrigel-coated transwells, incubated for 18 h to observe cell invasion, and recorded as corresponding photos. The data were from at least three reproducible independent experiments in which the raw invasive cell numbers were counted, and mean values with standard errors were plotted graphically. * Indicates *P* < 0.05 and ** indicates *P* < 0.01.

Several studies have highlighted the anticarcinogenic effect of Celecoxib on HCC cells; however, studies about mechanisms underlying the risk of HCC recurrence are limited. Therefore, we tested the effect of clinically available and suprapharmacological doses of Celecoxib on HCC cells and determined its effect in a chronic treatment module that mimicked long-term therapies. A cell invasion assay was employed to ascertain the oncogenic behavior of Tong, Huh 7, and HepG2 cells after treatment with suprapharmacological (100 µM, high-dose treatment) and clinically available (4 µM, low-dose treatment) doses of Celecoxib for 2 or 7 days. As shown in **Figure 2A**, exposure to a high-dose Celecoxib significantly reduced the cell invasion capability of HCC cells compared with vehicle-treated control cells in the 2-day treatment scheme. In a similar experiment, we did not observe any significant modulation in cell invasiveness in low-dose 2-day treated cells compared with controls (**Figure 2B**). However, metronomic Celecoxib treatment (4 µM, 7 days) significantly reduced the cell invasion capability of HCC cells (**Figure 2C**). These data indicated that low-dose Celecoxib treatment needs a longer time (i.e., metronomic therapy) to exert its effect against cell invasion of HCC.

**Figure 3:**
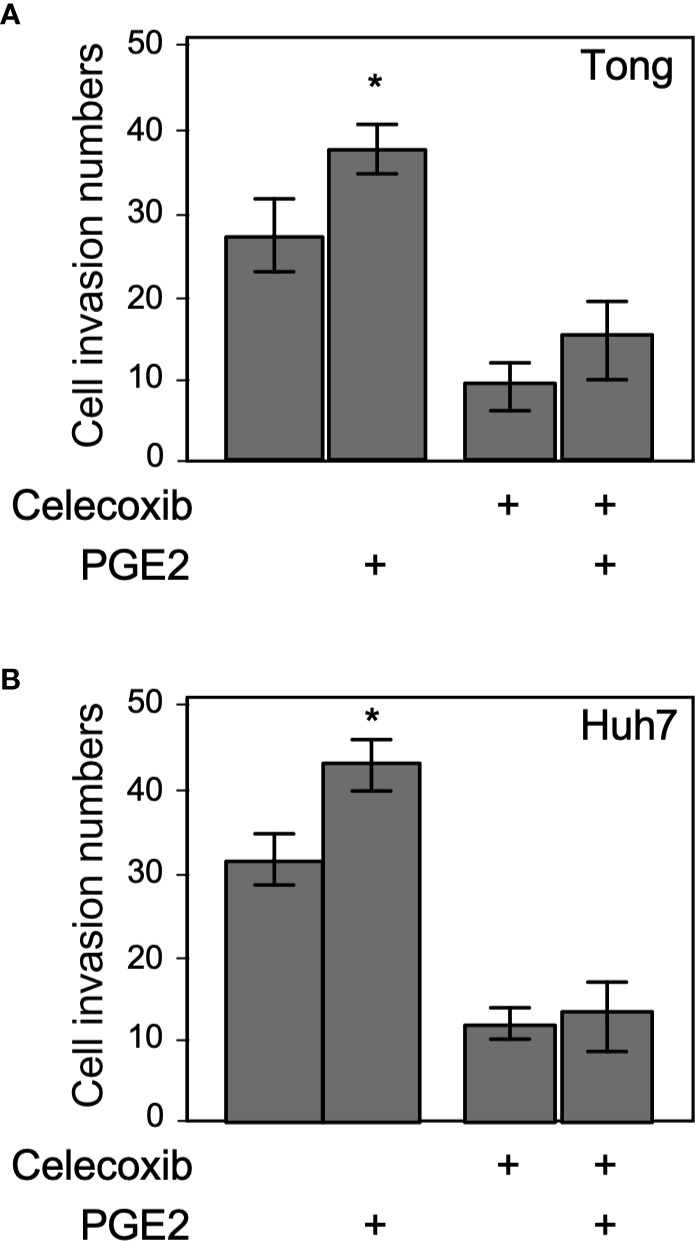
Suppression of invasion by metronomic Celecoxib treatments is a PGE2-independent event. **(A)** The cell invasion capacity of the Tong cells increased slightly by supra-physiological doses of PGE2 treatments (1 μM; lane 1 vs. 2). However, PGE2 co-treated with metronomic Celecoxib (4 μM, 7 days) did not reverse the Celecoxib suppression effect on cell invasion. **(B)** The cell invasion capacity of the Huh7 cells was increased by PGE2 treatments (1 μM; lane 1 vs. 2). However, PGE2 and metronomic Celecoxib cotreatment did not reverse Celecoxib-mediated suppression effect on cell invasion. The data were from at least three reproducible independent experiments in which the raw invasive cell number was counted, and mean values with standard errors were plotted on the graph. * Indicates *P* < 0.05.

Celecoxib is a selective inhibitor of COX-2, which generates PGE2 that stimulates cell invasion, proliferation, and migration behavior in hepatoma cells (22). Therefore, we tested the effect of metronomic Celecoxib treatment (4 µM, 7 days) on the invasive properties of HCC cells in the presence or absence of PGE2 (1µM, a supra-physiological concentration in the portal vein of the human) (23). As expected, PGE2 treatment significantly increased the invasiveness of HCC cells compared with vehicle-treated cells (**Figures 3A, B**). By contrast, a decline was observed in the invasion capability of HCC cells upon metronomic Celecoxib treatment when compared with vehicle-treated cells. However, stimulation with PGE2 did not significantly abrogate the anti-invasive effect of Celecoxib in HCC cells. These data indicated that metronomic Celecoxib inhibited basal as well as PGE2-stimulated cellular invasion, implicating the involvement of COX-2/PGE2-independent mechanisms in the suppression of invasive properties of HCC cells.

### Metronomic Celecoxib Suppressed the Invasive Properties of HCCs by Inhibiting MMP9 Through Perturbation of NFκB Activity

**Figure 4:**
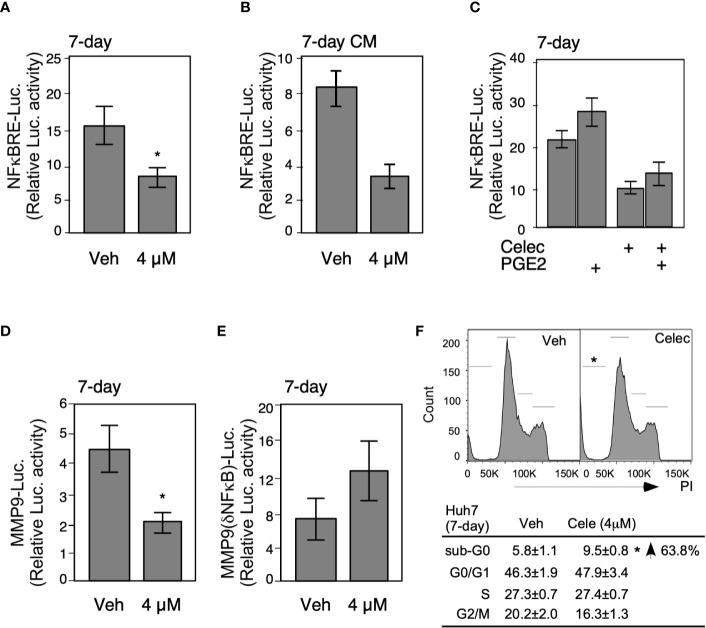
Metronomic Celecoxib treatments suppress HCC cell invasion through NFκB-MMP9 pathway. **(A)** The metronomic Celecoxib treatment suppressed NFκB response element (NFκBRE) activity in HepG2 cells. The HepG2 cells were treated with Celecoxib 4 μM for 4 days and transfected with a NFκBRE–luciferase construct; then, treatment was continued for another 3 days. The dual-luciferase activity was measured on the seventh day of treatments. **(B)** The conditioned medium from the HepG2 cells treated with metronomic Celecoxib suppressed NFκBRE activity. The conditioned medium obtained from the HepG2 cells treated with celecoxib (4 μM) for 7 days were used to treat the HepG2 cells containing the NFκBRE–luciferase construct to measure dual-luciferase activity. **(C)** PGE2 cotreatment did not rescue NFκBRE–luciferase activity inhibition caused by metronomic Celecoxib treatment. The HepG2 cells were treated with either Celecoxib 4 μM or PGE2 1 μM for 4 days, transfected with NFκBRE–luciferase construct, and treated again with Celecoxib or PGE2 for another 3 days. The dual-luciferase activity was measured at the seventh day of treatments. **(D, E)** The suppression of invasiveness by metronomic Celecoxib treatment could partially go through the NFκB-MMP9 pathway. Similar treatments [as **(A)**] were applied on the HepG2 cells, but transfected with MMP9 wild-type promoter (MMP9-luciferase; **(D)** construct, or NFκBRE deletion mutant of MMP9 promoter (MMP9–(ΔNFκBRE)–luciferase; **(E)** constructs to measure luciferase activity. The data were from at least four reproducible independent experiments in which the mean values with standard errors were plotted on graph. **(F)** The cell-cycle and sub-G0 population were measured in Huh7 cells treated w/wo metronomic celecoxib regimen. * Indicates *P* < 0.05.

To obtain more profound insights into the role of metronomic Celecoxib in NFκB-mediated invasiveness of HCCs, we assessed NFκB luciferase reporter activity after 7-day Celecoxib treatment. Results showed that low-dose (4 µM) Celecoxib treatment significantly suppressed the NFκB promoter activity (**Figure 4A**), whereas we observed a similar result upon analyzing CM for NFκB reporter activity (**Figure 4B**). Next, HCC cells were treated with Celecoxib in the presence or absence of PGE2 as performed previously and analyzed for NFκB promoter activity. As expected, PGE2 stimulation enhanced NFκB luciferase activity. However, Celecoxib inhibited both basal and PGE2-stimulated NFκB promoter activity (**Figure 4C**). These results suggest that metronomic Celecoxib treatment inhibited the invasive behavior of HCC cells through the suppression of NFκB transcriptional activity, and the mechanisms involved were independent of the COX-2/PGE2 pathway. Increased MMP9 expression is associated with enhanced tumor invasion properties; therefore, we ascertained the effect of Celecoxib on MMP9 promoter activity in HCC cells. We found that low-dose Celecoxib treatment significantly reduced MMP9 luciferase activity (**Figure 4D**). Because Celecoxib inhibited both NFκB and MMP9 activity, we speculated that the invasive properties of HCCs are mediated through NFκB transcriptional activity on the MMP9 promoter. To examine this possibility, we used an MMP9 luciferase reporter plasmid with a mutation at the NFκB binding site. Notably, MMP9 promoter activity with the mutated NFκB binding site was not affected by Celecoxib treatment (**Figure 4E**). Together, these data indicated that metronomic Celecoxib treatment exerted an inhibitory effect on the invasive property of HCC cells by reducing COX-2/PGE2 independent, NFκB-dependent MMP-9 expression. In addition to verify the metronomic cell growth inhibition effect through altering cell cycle or cell death, we performed PI staining following flow cytometry assay. As showed in **Figure 4F**, the G0/G1, S, and G2/M phases are comparable between vehicle or celecoxib treatment group (7 days). In terms of sub-G0 phase (represent as dead cells), the death population was increased in metronic celecoxib treated cells (**Figure 4F**).

**Figure 5:**
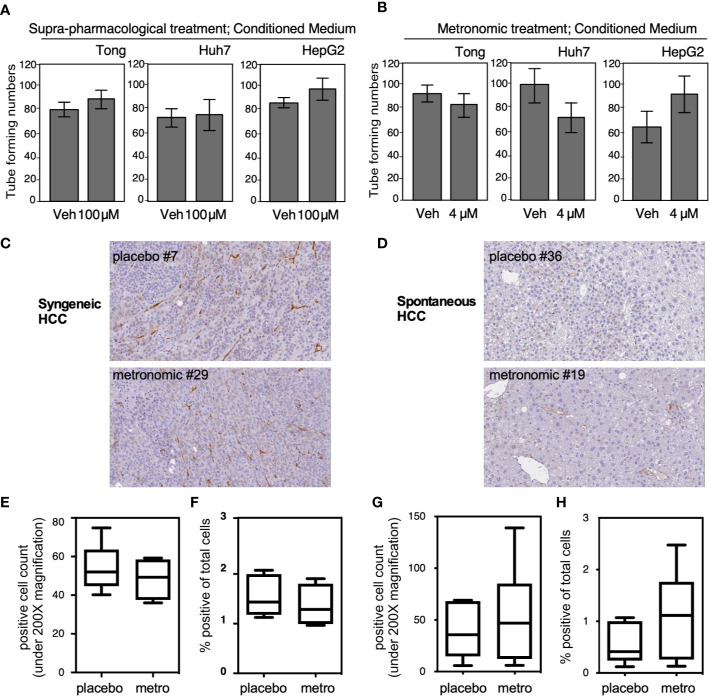
Microenvironmental influence from the HCC cells treated with metronomic Celecoxib did not alter the angiogenenesis phenotype. **(A, B)** Three HCC cell types (Tong, Huh7, and HepG2) were treated with suprapharmacological [**(A)** 100 μM for 2 days] or metronomic Celecoxib [**(B)** 4 μM, 7 days] and the conditioned medium (CM) was harvested. Each CM was then applied onto umbilical cord-derived endothelial cells to observe the angiogenic capacity of the CM. The tube-forming number was counted as described in materials & methods section, and quantitation result was plotted with standard error from three independent experiments. **(C, D)** Micro-vessel densities determined by CD34 IHC staining in comparable-sized HCCs from either syngeneic HCC models or spontaneous HCC models. Micro-vessel densities expressed by CD34+ cell counts **(E)** and percentage of CD34+ area to total scanned area **(F)** were comparable between the “metronomic Celecoxib” group and the placebo group in syngeneic HCC model. **(G, H)** a similar finding was also noticed in the spontaneous HCC model.

We examined tumor related angiogenesis by using the tube formation assay and CD34 IHC staining. We obtained CM from Tong, Huh 7, and HepG2 cells treated with a high dose (**Figure 5A**; 100 μM, 2 days) and low dose (**Figure 5B**; 4 μM, 7 days) of Celecoxib. Each CM was then applied onto umbilical cord-derived endothelial cells to observe the angiogenic capacity of the CM. Our results showed that CM obtained from cells treated with either high-dose or low-dose celecoxib could not significantly affect degree of angiogenesis compared to the placebo groups (**Figures 5A, B**). Similarly, CD34 IHC staining did not show significant difference of angiogenesis between the “metronomic Celecoxib” group and the placebo group in both syngeneic HCC and spontaneous HCC *in vivo* models (**Figures 5C–H**). All these findings indicated that the micro-environment of HCC treated by metronomic Celecoxib could not significantly affect HCC related angiogenesis.

### Metronomic Celecoxib Inhibited Cell Viability and Proliferation Capability of HCC Cells

**Figure 6:**
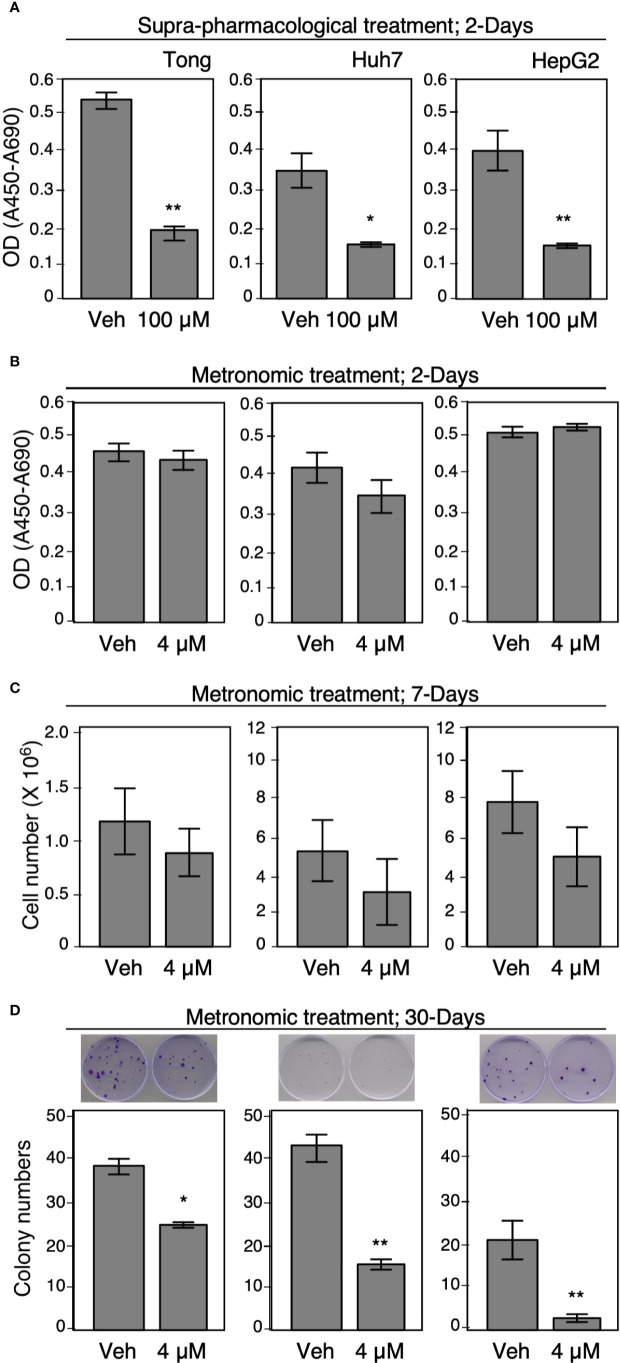
Metronomic Celecoxib treatments reduced HCC cell growth. **(A, B)** Suprapharmacological (100 μM) treatment, or clinically available Celecoxib treatment (4 μM) for 2 days affect human HCC cell growth (Tong, Huh7, and HepG2). The cells were seeded on 96-well plates, and the cell growth was measured by adding WST-1 reagent into the culture medium. After 1 h of incubation, the optical density or absorbance (OD or Å450-Å690) was recorded and the readings were plotted on graph. Unlike supra-pharmacological treatment, short-term clinically available Celecoxib (4 μM, 2 days) could not cause significant suppression on cell growth. **(C)** Metronomic Celecoxib (4 μM, 7 days) treatments on HCC cells could exhibit a greater suppression of HCC cell counts than did vehicle-treated control cells. The cells were plated onto 60-mm dish (2 × 10^5^ cells/plate) then treated with or without 4 μM Celecoxib, cultured for 7 days. The cell number was counted on day 7 by using plate cytometer, the total cell number was calculated, and the numbers were plotted on graph. **(D)** Long-term metronomic Celecoxib treatment (4 μM; 30 days) could reduce colony formation among the HCC cells. The HCC cells (500 cell/plate) were plated onto 60-mm dishes, treated with or without Celecoxib (4 μM), and were cultured for 30 days. The cells were fixed with 4% buffered formalin, stained with trypan blue, and recorded as corresponding photos. The colony numbers were counted, and the values were plotted on the graph. The data were obtained from at least three reproducible independent experiments, and the mean values with standard errors were plotted. * Indicates *P* < 0.05 and ** indicates *P* < 0.01.

To further delineate the effect of suprapharmacological and clinically available doses of Celecoxib treatment on HCC cell viability, we performed a series of colorimetric assays, cell viability assays, and colony formation assays for an incubation period of 2, 7, or 30 days, respectively. We found that a suprapharmacological dose (100 µM) of Celecoxib significantly inhibited HCC cell viability compared with control cells for a 2-day incubation period (**Figure 6A**). However, a similar treatment module at a clinically available dose (4 µM) did not elicit a significant suppression effect on HCC cell viability (**Figure 6B**). Next, we treated plated HCC cells with metronomic Celecoxib (4 µM, 7 days), and ascertained HCC cell numbers after treatment. Celecoxib-treated cells exhibited more significant suppression of HCC cell counts than did vehicle-treated control cells (**Figure 6C**). Next, we evaluated the effects of long-term metronomic Celecoxib treatment (4 µM, 30 days) on HCC cell proliferation potential that mimicked chronic HCC treatment modalities. The HCC cell colony formation ability was significantly attenuated over a long-term treatment duration (**Figure 6D**). Similar to metronomic Celecoxib against cell invasiveness, these data suggested the effects of Celecoxib at a clinically available dose in inhibiting HCC cell viability and proliferation may only be present when it is given for a longer time.

### Metronomic Celecoxib Inhibited the Cancer Stem/Progenitor Cells Phenotype in HCCs

**Figure 7:**
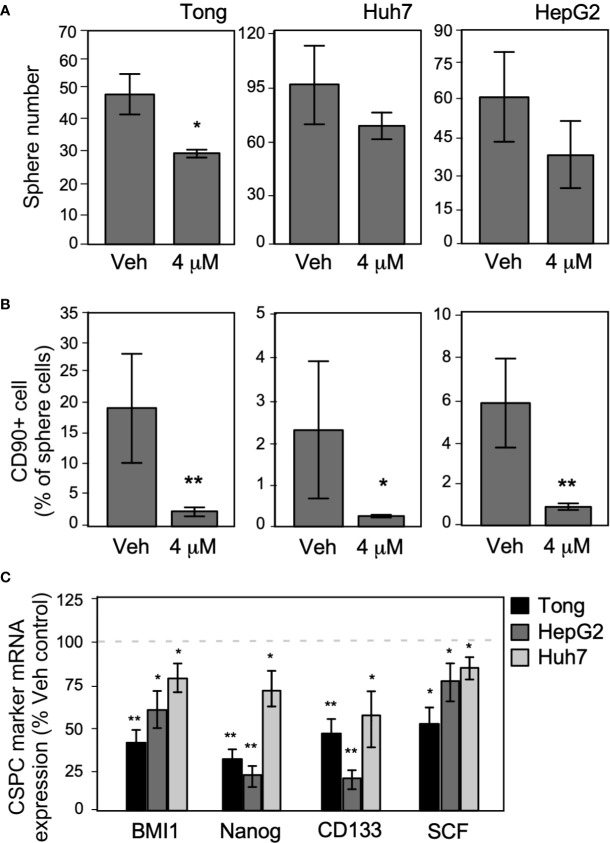
Long-term metronomic Celecoxib treatments could reduce CSPC self-renew capacity and CD90+ cell population in CSPC sphere cells. **(A)** Long-term metronomic Celecoxib treatments reduce HCC cells (Tong, Huh7, and HepG2) CSPC self-renewal. The HCC cells (500 cell/plate) were plated onto low-attachment 60-mm dishes with sphere-forming medium (low serum), treated with or without Celecoxib (4 μM; 21 days). The sphere number was counted, and the values were plotted on the graph. **(B)** Long-term metronomic Celecoxib treatments reduce CD90+ populations in the sphere cells. The cells from **(A)** were harvested, fixed with cold-methanol, stained with CD90 antibody, then observed CD90+ population by flow cytometry. The CD90+ percentage was plotted on the graph. **(C)** Long-term metronomic Celecoxib treatments reduce CSPC maker genes of the sphere cells. The cells from **(A)** were harvested, and the total RNA was extracted. CSPC marker genes expression (BMI1, Nanog, CD133, and SCF) was measured using real-time RT-PCR. The value was compared with vehicle treatment and plotted on the graph as % of Veh. control. The actin expressions were used as loading control of each set of experiments. The data were from at least three reproducible independent sets of the experiment, and the mean values with standard errors were plotted graphically. * Indicates *P* < 0.05 and ** indicates *P* < 0.01.

To test the effect of metronomic Celecoxib on the self-renewal potential of CSPCs, we examined its effect on the sphere formation ability of HCC cells and the marker expression of CSPCs. In the sphere formation assay performed using long-term metronomic Celecoxib treatment (4 µM, 21 days), Celecoxib significantly attenuated sphere formation in HCC cells (**Figure 7A**). Next, we assessed the expression level of the recurrence-associated stem cell marker CD90 in HCC sphere cells after metronomic Celecoxib treatment, as performed in previous experiments. We found that the number of CD90+ cells in the spheres was considerably lower among Celecoxib-treated cells than among vehicle-treated cells (**Figure 7B**). Finally, we determined the expression level of CSPC markers using Q-RT-PCR after metronomic Celecoxib treatment. The mRNA expression levels of BMI1, Nanog, CD133, and SCF were significantly lower in Celecoxib-treated HCC cells than in vehicle-treated HCC cells (p-values of CSPS markers: BMI1, Nanog, CD133 < 0.01, SCF < 0.05 in Tong cells; Nanog, CD133 < 0.01, and BMI1, SCF < 0.05 in HepG2 cells; BMI1, Nanog, CD133, SCF < 0.05 in Huh7 cells) (**Figure 7C**). By contrast, when we repeated similar exams while treating HCC cells at suprapharmacological concentrations, no viable cells could be detected after 21-day incubation time (data not shown).

## Discussion

**Figure 8:**
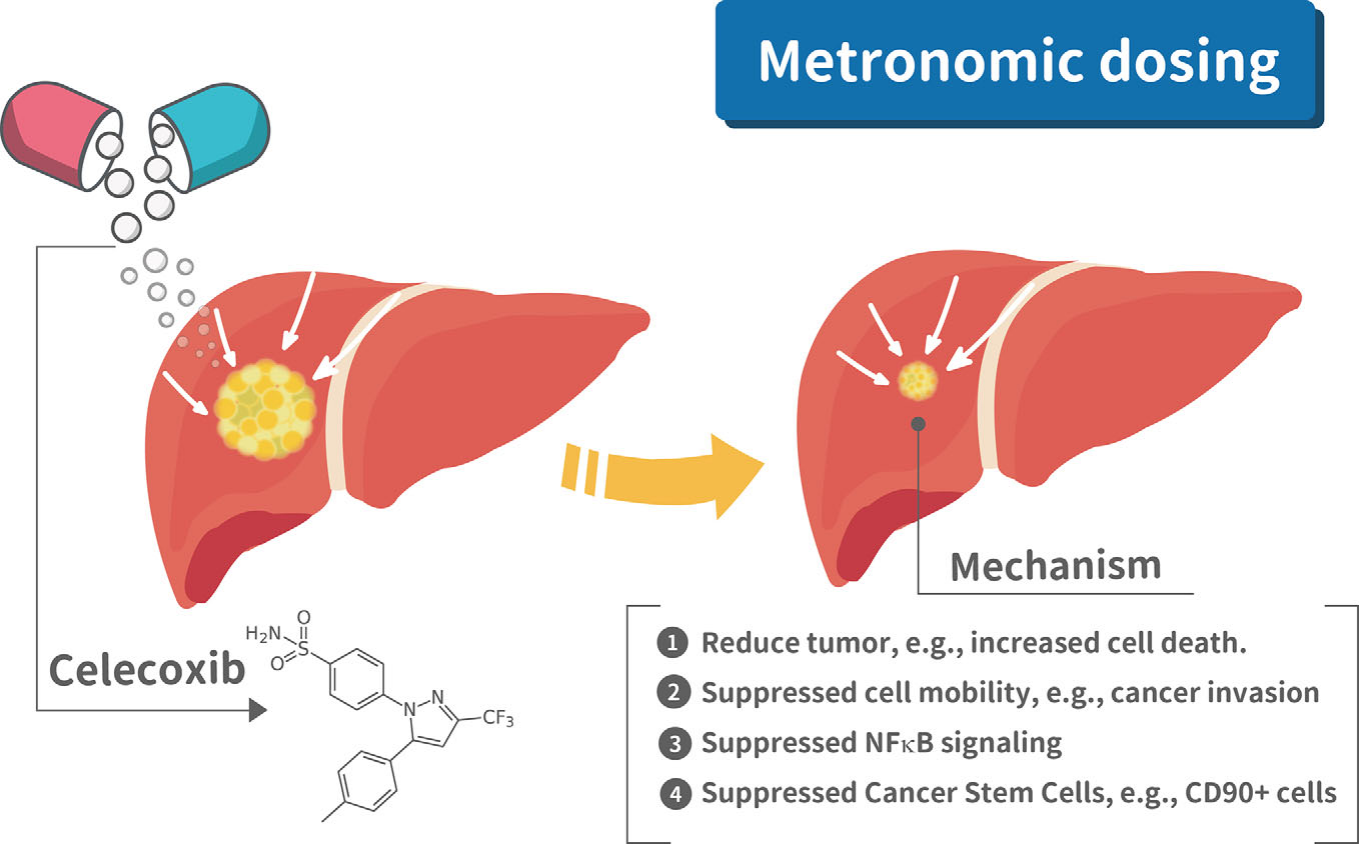
Mechanistic illustration of metronomic celecoxib effects on suppressing HCC prognosis. There are four mechanism for metronomic dosing in suppressing HCC prognosis, e.g., 1. Reduced tumor growth; 2. Suppressed cell mobility; 3. Suppressed NFkB signaling; 4. Suppressed cancer stem cells.

To our best knowledge, this is the first study to provide pre-clinical *in vivo* and *in vitro* evidence that metronomic Celecoxib at clinically available dosage significantly reduce HCC cell invasion, proliferation, stemness, and suppress tumor regrowth of seeded HCC (i.e., tertiary chemoprevention) and primary hepatocarcinogenesis (i.e., secondary chemoprevention). The mechanistic model of metronomic celecoxib on HCC suppression to prevent post hepatectomy surgery recurrence is illustrated in **Figure 8**. Besides, metronomic Celecoxib treatment mainly reduced HCC cell invasion via COX-2/PGE2 independent NF-kB/MMP9 dependent pathway. Based on these results, metronomic Celecoxib should be tried clinically as chemopreventive agents in selected high-risk HCC patients, such as HCC patients following curative treatments.

NSAIDs, regardless of selective or non-selective agents, are limited in clinically long-term usage due to increased risk of cardiovascular events (24). However, considering a significant risk of recurrent HCC after curative liver resection, some safer NSAIDs, such as selective COX-2 inhibitors, applied as chemopreventive agents in this high-risk population might be justified. Though some specific selective COX-2 inhibitors (e.g., Celecoxib) is a relatively safer medication than others (e.g., Rofecoxib) due to less risk of serious cardiac events, the cardiovascular risk still cannot be ignored and is significantly related to dose and dosing interval (24, 25). The cardiovascular risk in Celecoxib users was lowest for the 400-mg-QD dose compared to 200-mg-BID and 400-mg-BID (26). A pharmacokinetic study in a group of healthy subjects showed Cmax (705 ng/ml, equal to 1.85 µM) in those taking Celecoxib at a single dose of 200 mg (27). Therefore, we considered 4uM concentration of Celecoxib as a clinically available concentration while patients take Celecoxib at recommended doses (i.e., 400-mg-QD or 200-mg-BID). Regarding the dose of Celecoxib used at *in vivo* mice models, the conversion rate of drugs between human and mice is around 1 to 12 (28). Considering the risk of cardiovascular events in proportion to the dose of Celecoxib, we tried a dosage of Celecoxib (i.e., 10 mg/kg/d) at *in vivo* studies, and it is around 50-mg-QD Celecoxib in a 60-kg adult (24, 25). We considered that the reduced dose of Celecoxib should be safer for long-term application clinically as a chemopreventive medication. Hence, the chemopreventive effect and molecular mechanism of Celecoxib on HCC cells at a clinically available concentration is the most central and clinically relevant finding in this study.

Considering significant cardiovascular risk in high-dose Celecoxib use, we mainly exam the effect of metronomic Celecoxib (i.e., frequent administrating at a clinically available dose) on tumor invasion, proliferation, angiogenesis, and metastatic potential. Under metronomic Celecoxib treatment, tumor invasion, proliferation, and metastatic potential were significantly reduced. Our results corresponded well to the previous researches, where Celecoxib suppresses cell viability by inhibiting cell proliferation and colony formation, although previous researches mainly investigated Celecoxib at supra-pharmacological concentration (8). Unlike previous studies, angiogenesis was not significantly attenuated under metronomic Celecoxib treatment (8). A similar finding was also noticed by measuring micro-vessel density by CD34 IHC staining at *in vivo* models. The results indicated that anti-carcinogenic effect of metronomic Celecoxib may not rely on anti-angiogenesis effect.

In the pre-clinical *in vivo* study, we investigated the effect of metronomic Celecoxib on *in vivo* tumor growth of HCC with either homogenous or heterogeneous genetic backgrounds using two different animal models. We found a significant reduction in tumor regrowth of seeded syngeneic HCC while treating with metronomic Celecoxib compared to placebo. The implanted Hepa1-6 HCC cell line is derived from C57L mice with homogenous genetic background and widely accepted for studying *in vivo* tumor growth and metastasis of HCC in immunocompetent environment (29). However, syngeneic implanted HCC model using an established HCC cell line after multiple passages may not truly reflect clinically relevant situations. Thus, we used the other animal model (i.e., HBVtg-HCC model) to investigate spontaneous hepatocarcinogenesis that comes from freshly developed HCC tumor cells with heterogeneous genetic backgrounds. Noteworthy, Celecoxib had been proven effective in chemoprevention in the DEN-induced HCC animal model if it is given before or along with DEN (200mg/Kg) because Celecoxib may upregulate cytochrome-P450 activity and reduce the toxicity of DEN sequentially (30). However, this model is not the case related to the clinical situation that exposure to a carcinogen (e.g., hepatitis virus B or aflatoxin) usually precedes the usage of chemopreventive drugs. In our study, metronomic Celecoxib was given long (at the age of 20^th^ week) after low-dose DEN (20mg/Kg, at the age of 2^nd^ week) administrated to HBVtg mice. This model is more clinically relevant and more like secondary chemoprevention to reduce progression to HCC from underlying chronic viral hepatitis (2).

NF-κB has been well known as a cancer promoter, particularly in inflammation-associated tumor such as HCC (31). The mechanism of anti-carcinogenic effect by Celecoxib (e.g., inhibition on NFκB) were extensively investigated (8). However, most studies investigated the interaction between Celecoxib and NFκB at clinically irrelevant conditions, such as supra-pharmacologic dosage of Celecoxib (i.e., more than five µM) or short-term treatment (e.g., hours) (8). To determine the exact mechanism under clinically relevant situations, we particularly treat the Luciferase system using metronomic Celecoxib (4 µM, 7 days). Consistent with previous reports, we found that NFκB transcriptional activity could also be suppressed in HCC cells by metronomic Celecoxib treatment (8). Furthermore, we could not abrogate the inhibitory effect of metronomic Celecoxib on NF-κB even by applying supra-physiological dosage of PGE2 (1µM) (23), and it implied that metronomic Celecoxib mainly exerts its inhibitory effect via NFκB dependent and COX2/PGE2 independent pathway.

We examined whether metronomic Celecoxib treatment could suppress the more resistant subpopulations of HCCs by reducing the numbers of sphere-forming cells or CSPCs. CSPCs have extensive self-renewal ability, tumorigenesis, and differentiation potential; consequently, they give rise to new anaplastic tumor cells that exhibit resistance to cytotoxic chemotherapy and ionizing radiation (32, 33). This resistance may be attributed to their presumably slow cell cycle and overexpression of efflux pumps (34), which gives rise to CSPC subpopulations within each tumor (19, 32, 33, 35). Given the essential role of CSPC in metastasis, recurrence, and therapeutic resistance, it becomes imperative to identify novel therapies, specifically targeting CSPCs, which can potentially eradicate the renewal capacity of the tumor (36). In this study, we found that a metronomic Celecoxib therapy could significantly reduce sphere formation in HCCs, CD90+ population in sphere cells, and expression of the CSPC markers (BMI1, Nanog, CD133, and SDF). The finding suggested that metronomic Celecoxib treatment could reduce the formation and phenotype of CSPC in HCC that also corresponded to the previous study that Celecoxib could suppress HCC stemness at a higher-than-normal concentration (10µM) (9).

This study evaluated the invasiveness, cell proliferation, metastatic potential, and tumor growth of HCC cells under metronomic Celecoxib treatment using *in vivo* and *in vitro* system. Because of cardiovascular risk and effective anti-carcinogenesis of selective COX-2 inhibitors, we considered metronomic Celecoxib therapy might be a potentially effective chemopreventive agent for reducing the risk of tumor recurrence, progression, and metastasis in selected high-risk HCC patients such as HCC patients after curative treatments. Based on this pre-clinical *in vivo* and *in vitro* study, further pharmacokinetic studies and clinical studies are warranted to validate the effective dose and chemopreventive potential of metronomic Celecoxib against HCC.

## Data Availability Statement

The raw data supporting the conclusions of this article will be made available by the authors, without undue reservation.

## Ethics Statement

The animal study was reviewed and approved by China Medical University Committee of Laboratory Animal Welfare.

## Author Contributions

C-C Y performed the experiments, developed the concept, and manuscript editing. SP, P-Y L, and S-Y Y conducted experiments and interpreted data and drafted the manuscript. H-C L, L-B J, and W-C C were responsible for clinical consultation and participated in manuscript editing. W-L M developed the concept, supported the entire study, and edited and approved the final version of the manuscript. All authors contributed to the article and approved the submitted version.

## Funding

This study was supported in part by grants from the Taiwan Ministry of Sciences and Technology (MOST 107-2314-B-039-011; MOST 108-2320-B-039-017; MOST 108-2314-B-039-043-MY3; MOST 108-2314-B-039-052; MOST 109-2327-B-039-002); National Health Research Institution (NHRI-EX109-10705BI); and China Medical University/Hospital (DMR-CELL-1810; DMR-CELL-1907; DMR-107-033; DMR-108-080; DMR-108-179; DMR-109-240, DMR-109-019, and DMR-109-201; CMU108-MF-33; CMU106-S-28).

## Conflict of Interest

The authors declare that the research was conducted in the absence of any commercial or financial relationships that could be construed as a potential conflict of interest.
