# Supplementary figures and images for: Metronomic Celecoxib Therapy in Clinically Available Dosage Ablates Hepatocellular Carcinoma via Suppressing Cell Invasion, Growth, and Stemness in Pre-Clinical Models
Source: Front Oncol. 2020 Oct 21;10:572861. doi: 10.3389/fonc.2020.572861 (PMC7609882; doi:10.3389/fonc.2020.572861)

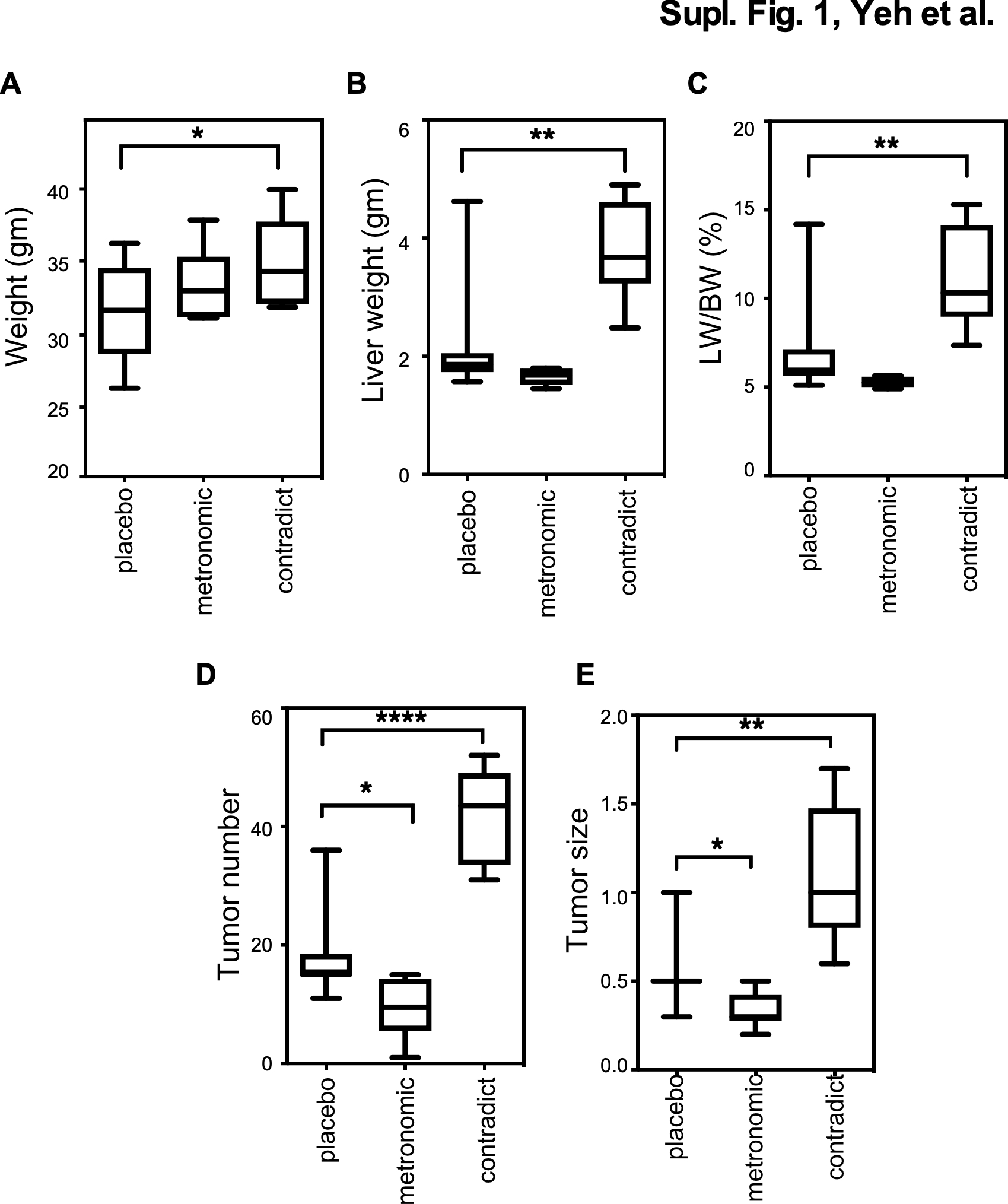

Supplement: Supplementary file 1 [file Image_1.jpeg]
