# Supplementary material for: Metronomic Celecoxib Therapy in Clinically Available Dosage Ablates Hepatocellular Carcinoma via Suppressing Cell Invasion, Growth, and Stemness in Pre-Clinical Models
Source: Front Oncol. 2020 Oct 21;10:572861. doi: 10.3389/fonc.2020.572861 (PMC7609882; doi:10.3389/fonc.2020.572861)
Supplement: Supplementary file 2 [file Table_1.docx]

**Supplemental Materials and Methods**

**Cell culture and reagents**

HepG2, Huh7, and Tong/HCC cell lines were gifted by Dr. Jou YS (Academia Sinica, Taiwan) and were maintained as published elsewhere.^1^ In brief, HCC cells were cultured in Dulbecco’s modified Eagle medium (DMEM, Invitrogen) supplemented with 10% fetal calf serum (FCS), 1% glutamine, and 1% penicillin/streptomycin. The following chemicals were used: PGE2 (Sigma–Aldrich, CA, USA), Celecoxib (Sigma–Aldrich), formaldehyde (Avantor, PA, USA), and agarose 1 (AMRESCO, OH, USA). The sphere medium included DMEM (GIBCO, CA, USA), B-27 supplement (GIBCO, USA), insulin and human recombinant zinc solution (4 mg/mL) (GIBCO), EGF (SIGMA, MO, USA), DMEM (GIBCO), fetal bovine serum (FBS; GIBCO), methanol (Avantor), and triton (BIO BASIC Inc, NY, USA). The antibodies used for immunohistochemistry study were CD34 (abcam, ab81289).

**Cell invasion assay**

To test the effect of Celecoxib on HCC cell mobility, we performed a transwell cell invasion assay as previously published.^2^ HCC cells were pre-treated with Celecoxib in different situations (e.g., the supra-pharmacological dose for two days, or clinically available dose for 2 or 7 days). In cells that were cultured more than 3 days, we exchanged culture medium with or without Celecoxib every three days. After pre-treatment, we harvested 3×10^4^ cells and seeded them in matrigel (Invitrogen)-coated transwell (Corning, NY) plates and allowed them to migrate for 18 hours. At the end of incubation, we scrapped out the cells in the inner layer of the chamber, and added trypan blue to stain cells in the outer chamber; counted stained cells under a light microscope (Nikon, Eclipse 80i). The number of cells invaded was counted in three random areas of each experiment and quantified.

**Conditioned medium (CM)**:

The growth factors and cytokine within the microenvironment of HCC cells could be implicated in the regulation of tumor growth, invasion, and angiogenesis ^3, 4^. Therefore, to mimic the micro-environment of HCC under the treatment of Celecoxib, we used CM as a substitute for pure Celecoxib to investigate angiogenesis and molecular mechanism. HCC cells (Tong or Huh7 or HepG2) were seeded (1 x 10^5^ cells) in a 10-cm dish. The next day, the medium was replaced with 10 mL of fresh medium (DMEM with 10% FBS) and treated with four μM or 100 μM Celecoxib and incubated for 48 h. Then, the medium was filtered using a 0.22-μM filter, collected, and stored at −20°C for future use.

**Luciferase assay**

The plasmids pGL3-NFκBRE and pGL-MMP9 Luc reporter gene were gifts from Dr. Edward M. Schwarz (University of Rochester Medical Center, Rochester, NY), and the results were published earlier.^5^ Briefly, 4 × 10^4^ HepG2 cells were plated on 12-well plates 24 h before transfection. For plasmid transfection, lipofectamin (Invitrogen) was used following the instruction manual; then, cells were exposed to Celecoxib at indicated concentrations and time points, as shown in figure 3. At the end of the treatment, cells were harvested, and the cell lysates were analyzed for the luciferase reporter activity by using a dual-luciferase reporter assay system (Promega).

**Tube formation assay**

We measured the potential effect of Celecoxib on cancer-related angiogenesis, as described in a previous study.^2^ Matrigel (BD Biosciences) was dissolved at 4°C overnight, and 48-well plates were prepared by coating each well with 100 μL of matrigel and incubating them at 37°C for 30 min. EPCs (Endothelial Progenitor Cells; 6 × 10^4^) were cultured in 200 μL of medium, which also included 50% EGM-MV2 medium (Promo cell, Germany) and 50% conditioned medium (CM). After 8 h of incubation at 37°C, EPC tube formation was assessed using a photomicroscope, and each well was photographed at 200× magnification under a light microscope. The total number of tubes and the total number of tube branches were calculated using MacBiophotonics Image J software (Bethesda, MD, USA).

**Cell viability and cell counting analysis**

The effect of Celecoxib on cell viability after 2-days treatment was determined using the WST-1 assay (Roche, USA), as reported previously.^6^ In brief, 10^3^ three HCC cells/100 μL/well were seeded in 96-well plates with DMEM in 10% FBS and incubated with Celecoxib at selected concentrations for designated periods. Then, 10 μL of WST-1 solution was added to each well, and cells were incubated for one h. Next, we determined cell viability by measuring the absorbance proportional to the relative abundance of live cells in the given wells by using colorimetric detection in an enzyme-linked immunosorbent assay plate reader (Beckman Coulter Paradigm ^TM^ Detection Platform) at 450 and 690 nm. We investigated short-term cell growth by cell counting assay that 10^3^ cells were cultured onto 6-cm dishes with treatments for 7-days. At the time of harvest, the cells were detached and subjected to Cell Counter (BD Bioscience, Taipei, Taiwan). We used trypan-blue staining to exclude dead cells.

**Colony formation assay for long-term cell growth observation**

We tested the effect of long-term (30 days; 200 cells/dish) metronomic Celecoxib treatment on HCC cell growth by using the colony formation assay, as described previously.^2^ In brief, we seeded HCC cells onto 6-cm culture dishes and allowed them to attach for 24 h. Then the fresh medium was added with or without Celecoxib every three days consecutively, and the cells were incubated for 30 days. At the end of indicated time points, 1 mL of 4% formaldehyde was added to fix cells and incubated at room temperature for 1 h. Cells were stained using crystal violet. After 1 h, crystal violet was washed from the cell culture dish, and colonies were photographed and counted.

**Sphere formation assay**

To examine the effects of Celecoxib on the self-renewal of CSPCs, we performed the sphere formation assay following a previously described protocol.^7^ In short, we seeded cells (Tong, Huh7, and HepG2; 5 × 10^2^ cells/dish) onto 6-well plates precoated with 1% agarose and maintained in 5 mL of sphere medium (DMEM; B-27, 1X; insulin, 5 μg/mL; and EGF, 20 ng/mL) for 21 days. Every 2–3 days, we added 500 μL of fresh medium. We photographed cells and counted cell colonies.

**Flow cytometry for CD90 detection**

Cells were harvested into a tube and allowed to stand at room temperature for 10–15 min. Cells were pelleted through centrifugation at 800 rpm for 10 min at 4°C, and the supernatant was discarded carefully. The pellet was completely suspended in 300 μL of phosphate-buffered saline (PBS); then, 700 μL of methanol was added and cells were allowed to stand for 10 min to enable fixation. Cells were divided into four groups (nonstaining, IgG, DMSO-CD90, and celecoxib-CD90) in flow tubes and washed using PBS. Cells were pelleted through centrifugation at 1500 rpm for 5 min at 4°C, and the supernatant was discarded. Next, 500 μL of 0.2% Triton and 500 μL of PBS were added, and the mixture was allowed to stand for 10 min. Cells were washed and centrifuged, and the supernatant was discarded. Next, 200 μL of 5% BSA was added to cells to block them and incubated for 30 min. Cells were washed and centrifuged, and the supernatant was discarded. Next, 200 μL of 5% BSA and 2 μL of antibody solution were added to cells; then, cells were incubated for 30 min at 4°C and protected from light. The washing step was repeated twice, and the supernatant was discarded between washes. Stained cells were suspended in 300–500 μL of PBS (depending on the number of cells). Data were acquired using a flow cytometer.

**Quantitative real-time polymerase chain reaction analysis**

The mRNA expression of the CSPC marker gene was examined through quantitative real-time polymerase chain reaction (Q-RT-PCR) following a protocol described earlier. ^8^ Whole cellular RNA extracts were prepared from cultured cells by using TriZol (Invitrogen) and the phenol–chloroform pH 6.7/8.0 (AMRESCO, Solon, Ohio) method. Two microgram of total RNA was used for reverse transcription by using PrimeScript and TaKaRa kit (TaKaRa, Japan). The messenger RNA (mRNA) expression levels of CSPC markers (SCF, Bmi1, Nanog, and CD133) were determined through Q-RT-PCR by using C1000 Cycler CFX96 Real-time System (BioRad) with iQ SYBR Green Supermix (BioRad). Table 1 illustrates the primers used for Q-RT-PCR determination. Relative gene expressions were determined by normalizing the expression level of the target gene to the expression level of housekeeping genes (U6). Threshold value (Ct) dynamics were used (2^-ΔΔCt^) for quantification of gene expression.

**References**

1. Hung YC, Chang WC, Chen LM, et al. Non-genomic estrogen/estrogen receptor alpha promotes cellular malignancy of immature ovarian teratoma in vitro. *J Cell Physiol* 2014; 229(6):752-61.

2. Chung WM, Chang WC, Chen L, et al. Ligand-independent androgen receptors promote ovarian teratocarcinoma cell growth by stimulating self-renewal of cancer stem/progenitor cells. *Stem Cell Res* 2014; 13(1):24-35.

3. Zhu AX, Duda DG, Sahani DV, et al. HCC and angiogenesis: possible targets and future directions. *Nat Rev Clin Oncol* 2011; 8(5):292-301.

4. Novikova MV, Khromova NV, Kopnin PB. Components of the Hepatocellular Carcinoma Microenvironment and Their Role in Tumor Progression. *Biochemistry (Mosc)* 2017; 82(8):861-873.

5. Ma WL, Hsu CL, Yeh CC, et al. Hepatic androgen receptor suppresses hepatocellular carcinoma metastasis through modulation of cell migration and anoikis. *Hepatology* 2012; 56(1):176-85.

6. Chung WM, Chang WC, Chen L, et al. MicroRNA-21 promotes the ovarian teratocarcinoma PA1 cell line by sustaining cancer stem/progenitor populations in vitro. *Stem Cell Res Ther* 2013; 4(4):88.

7. Chen L, Bao BY, Chang WC, et al. Short androgen receptor poly-glutamine-promoted endometrial cancer is associated with benzo[a]pyrene-mediated aryl hydrocarbon receptor activation. *J Cell Mol Med* 2018; 22(1):46-56.

8. Zhang H, Cheng S, Zhang M, et al. Prostaglandin E2 promotes hepatocellular carcinoma cell invasion through upregulation of YB-1 protein expression. *Int J Oncol* 2014; 44(3):769-80.
